# Optimized Mouse Model of Sepsis‐Associated Encephalopathy: A Rational Standard Based on Modified SHIRPA Score and Neurobehaviors in Mice

**DOI:** 10.1111/cns.70365

**Published:** 2025-04-09

**Authors:** Yuewen Xin, Mi Tian, Xu Pei, Shuixiang Deng, Yao Wang, Feng Zhao, Thomas Behnisch, Yanqin Gao, Ye Gong

**Affiliations:** ^1^ Department of Critical Care Medicine of Huashan Hospital, State Key Laboratory of Medical Neurobiology, MOE Frontiers Center for Brain Science and Institutes of Brain Science Fudan University Shanghai China; ^2^ Department of Neurosurgery, Huashan Hospital Fudan University Shanghai China; ^3^ National Center for Neurological Disorders, Huashan Hospital Fudan University Shanghai China

**Keywords:** behavioral tests, cognitive deficits, long‐term potentiation, modified SHIRPA score, sepsis‐associated encephalopathy

## Abstract

**Background:**

Sepsis‐associated encephalopathy (SAE), a severe neurological disorder, is marked by widespread brain dysfunction. At present, there is no universally accepted criterion for diagnosing SAE in animal models. This study proposes a standardized evaluation method for SAE in mice, addressing inconsistencies in current research.

**Method:**

Using a cecal ligation and puncture (CLP) model to induce sepsis, we assessed the physiological status of mice with a modified SHIRPA score to differentiate SAE from non‐SAE, validating our findings through various behavioral tests and evaluations of neuroinflammation and neuronal damage.

**Results:**

Our findings revealed that the conventional mild–moderate–severe categorization of SHIRPA was insufficient for distinguishing between SAE and non‐SAE. To enhance differentiation, we classified mice based on the median modified SHIRPA score, validating this approach through behavioral tests including the Y‐maze, three‐chamber social test, and open field test. This method effectively identified neurological impairments in septic mice. Further validation involved assessing neuronal damage, neuroinflammation, the Morris water maze, and long‐term potentiation (LTP) in the hippocampal CA1 region. Results indicated that mice in the up‐Median group exhibited greater neuroinflammation, neuronal injury, and cognitive deficits compared to the down‐Median group.

**Conclusions:**

This study establishes a reliable evaluation method for SAE in murine models, facilitating improved differentiation between SAE and non‐SAE. Such advancements will enhance our understanding of the pathogenesis of SAE and guide more effective treatment strategies.

## Graphical abstract

This study focuses on the standardized establishment of animal models for sepsis‐associated encephalopathy (SAE). We employed a novel scoring method to identify mice with SAE, which may provide a potential theoretical foundation and research direction for future studies on SAE.

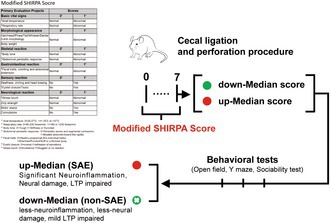

**Abbreviations**
aCSF: artificial cerebrospinal fluid
ANOVA: analysis of variance
AUC: area under the curve
CA: cornu ammonis
CD: cluster of differentiation
CLP: cecal ligation and puncture
CSS: clinical severity score
CTX: cerebral cortex
EEG: electroencephalogram
EPSP: excitatory postsynaptic potentials
fEPSP: field excitatory postsynaptic potentials
Iba 1: ionized calcium binding adaptor molecule 1
LPS: lipopolysaccharide
LTP: long‐term potentiation
M‐CASS: mouse clinical assessment score for sepsis
MSS: murine sepsis score
NeuN: neuronal nuclear antigen
ROC: receiver operating characteristic
SAE: sepsis‐associated encephalopathy
SD: standard deviation
SHIRPA: The SmithKline, Harwell, Imperial College, Royal Hospital, Phenotype Assessment
SIRS: systemic inflammatory response syndrome

## 1 Introduction

Sepsis is a life‐threatening organ dysfunction caused by the body's dysregulated response to infection, associated with high mortality and morbidity rates in both hospitalized and critically ill patients [1]. A recent global analysis estimated that sepsis was responsible for 11 million deaths in 2017, accounting for 19.7% of all deaths worldwide, representing a significant global health burden, particularly in sub‐Saharan Africa, Oceania, and parts of Asia [2]. Several animal models have been developed to better understand the pathophysiology of sepsis. These models include the injection of toxic agents (such as lipopolysaccharide (LPS), endotoxins, or zymosan), the injection of viable pathogens (such as bacteria or intestinal contents), and the disruption of tissue barriers (e.g., intestinal perforation or wound sepsis models) [3, 4]. Among these, polymicrobial sepsis induced by cecal ligation and puncture (CLP) is the most commonly used model, as it closely mimics the progression and characteristics of human sepsis [5].

Sepsis‐associated encephalopathy (SAE) is a severe neurological syndrome characterized by diffuse brain dysfunction [6]. More than half of hospitalized patients with sepsis and systemic inflammatory response syndrome (SIRS) develop SAE, which is associated with increased mortality, cognitive impairment, and focal neurological deficits [7, 8]. While SAE has traditionally been considered a fully reversible condition, increasing evidence from animal experiments and clinical studies suggests that sepsis may cause structural brain injury and lead to long‐term neurological sequelae [9, 10]. Like sepsis, SAE is treatable, and timely implementation of targeted interventions can improve patient outcomes [11]. Early recognition and diagnosis of SAE are crucial for effective management, as SAE is known to involve complex pathophysiological changes, and there is currently no consensus on the precise mechanisms or targeted pharmacological treatments [12]. Animal models play a key role in the investigation of SAE etiology and potential therapies. Among the aforementioned methods, CLP and LPS injection are the most frequently employed to establish models of SAE [13]. CLP mimics polymicrobial infections, making it a more physiologically relevant sepsis model, although it requires surgical expertise and has a higher mortality rate. In contrast, LPS injection is simpler and allows rapid induction of inflammation, but lacks the complexity and long‐term relevance of actual sepsis [14].

The success of sepsis animal models can be assessed through clinical signs, microbiological confirmation, histopathological examination, survival rates, and physiological parameters [15]. Scoring systems, such as the Mouse Clinical Assessment Score for Sepsis (M‐CASS) and the Murine Sepsis Score (MSS), are commonly used to assess disease severity and prognosis [13, 16]. The SHIRPA (SmithKline Beecham, Harwell, Imperial College, Royal London Hospital, Phenotype Assessment) score, first proposed by D.C. Rogers and colleagues in 1997, includes over 40 scoring items covering sensory responses, motor function, behavioral characteristics, and neurological abnormalities in mice [17, 18]. Recently, an increasing number of researchers have used the modified SHIRPA score to assess sepsis‐associated brain injury, or SAE [19, 20]. This score is valuable for assessing behavioral changes in mice following infection or inflammation, providing insight into how sepsis impacts the nervous system and overall health of the animals. Some studies assume that successful modeling of sepsis equates to successful modeling of SAE, while others use the clinical severity score (CSS) or delta waves in the EEG as inclusion criteria for identifying SAE in mice [10, 21]. However, there is currently no universal and widely accepted standard for identifying animal models of SAE, highlighting the urgent need for a standardized system for developing and analyzing SAE.

It is acknowledged that not all patients with sepsis experience severe acute events, yet SAE occurs in more than half of these patients. Similarly, in animal models of sepsis, some animals do not develop SAE. Therefore, there is an urgent need for an effective evaluation method and standard to assess the degree of neuroprotection in SAE. In current research on neuroprotection in SEA, animal models of SAE typically use animals with a modified SHIRPA score > 3, with the assumption that these animals have developed SAE. However, there is no clear evidence as to whether animals with a modified SHIRPA score > 3 have neurological deficits. In fact, the modified SHIRPA score gradually decreases within 1–7 days after surgery leading to imprecise classification of SEA animal models.

In this study, we hypothesized that the Median of the modified SHIRPA score could serve as a criterion for SAE. We continuously assessed mice post‐CLP using the modified SHIRPA score, filtering out those with significant brain dysfunction by neurological assessment. As neurobehavioral scores, neurobehavioral tests, and electrophysiological techniques are commonly used in the analysis of SAE models [13, 22], we further validated the accuracy of this criterion using neurobehavioral scores, behavioral tests (open field test, three‐chamber social test, Y‐maze, Morris water maze), and electrophysiological recordings of field EPSP and LTP in isolated brain tissue. Our study aims to provide a reliable clustering method for the study of pathological mechanisms in SAE, allowing researchers to consistently and systematically gain insight into key functional aspects of SAE pathology.

## 2 Materials and Methods

### 2.1 Animals

Wild‐type adult male C57BL/6J mice (purchased from SPF (Beijing) Biotechnology Co. Ltd.) were used as experimental subjects in this study. All animals were maintained at a weight of 22–30 g (6–8 weeks old) prior to the experiment. The mice were housed in transparent cages at 22°C–25°C under a 12‐h light/dark cycle, with free access to food and water. All experiments were approved by the Ethics Committee of Fudan University Huashan Hospital (approval number 2024‐HSYY‐635, Shanghai, China). The experiments were conducted in accordance with the National Institutes of Health Guide for the Care and Use of Laboratory Animals. All animal experiments were reported in compliance with the ARRIVE guidelines.

### 2.2 Cecal Ligation and Perforation Procedure

Mice were anesthetized with isoflurane. The mice were positioned supine, oxygenated via a mask, and their breathing was controlled by an animal ventilator (respiration ratio set at 1:1; tidal volume set to 2–4 mL; breathing rate set to 60 breaths/min). The mice inhaled a gas mixture of 30% O_2_ and 70% N_2_ mixed with 1%–2% isoflurane to maintain anesthesia during surgery. Sepsis was induced in the mice via CLP. Under sterile conditions, a 1.5–2 cm incision was made along the midline of the abdomen, and the fascia of the abdominal muscle was gently removed. The cecum was located, isolated, and pulled outside the abdomen. Following the procedure described by Rittirsch et al. [23], the ileocecal valve was located, and a median cecal ligation and puncture was performed. The sham group omitted the ligation and puncture steps. Postoperatively, the mice were administered 5 mL/100 g (37°C) isotonic saline for fluid resuscitation. They were placed on a temperature‐controlled heating pad to maintain their body temperature. Once the mice regained consciousness, they were returned to their cages and relocated to an animal housing facility with controlled temperature and humidity.

### 2.3 Modified SHIRPA Score

**FIGURE 1:**
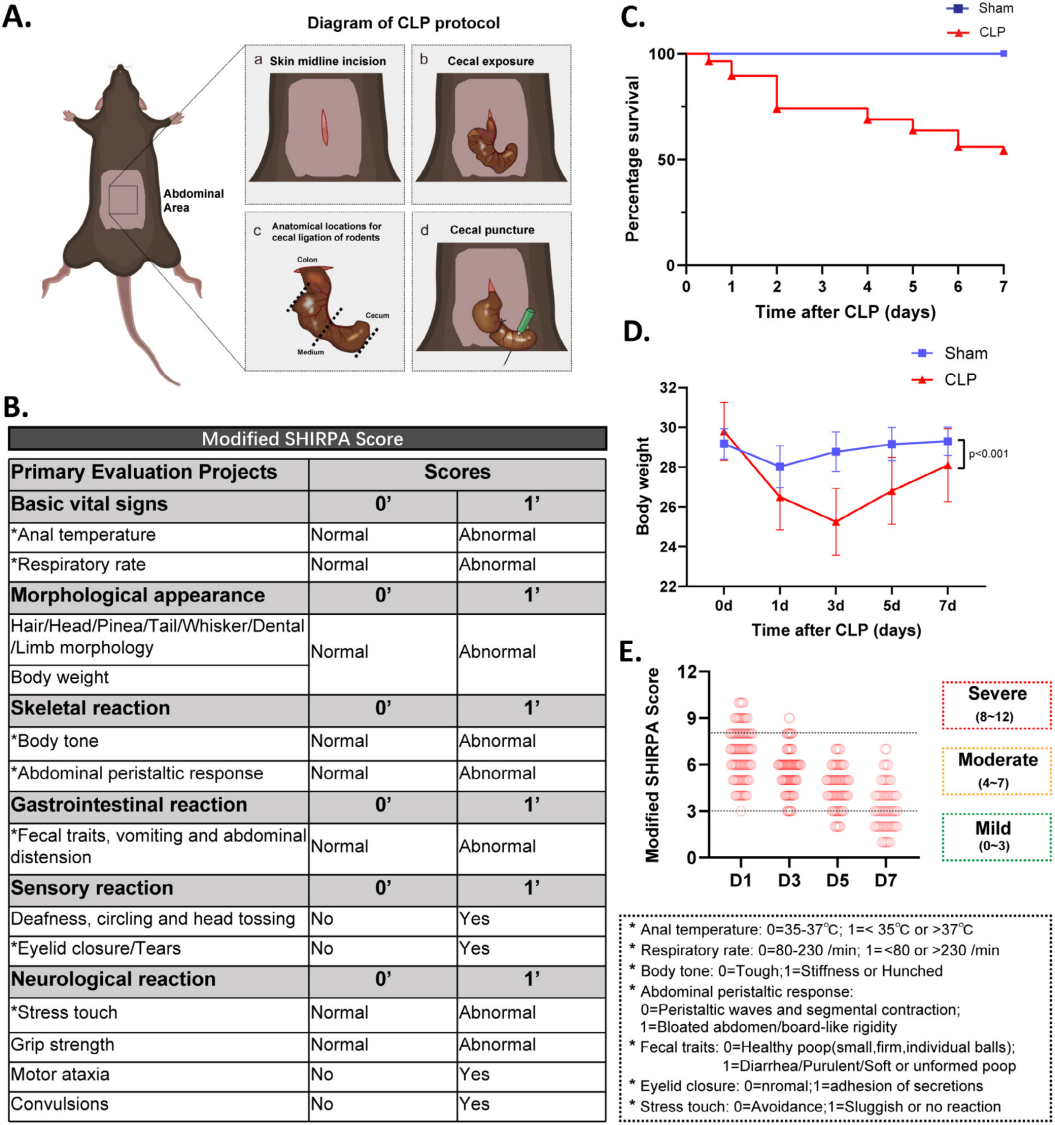
Modified SHIRPA Score, body weight, and the survival rate of CLP mice. (A) Schematic diagram of CLP surgery. (B) The modified SHIRPA scores protocol. (C) The survival rate within 7 days after CLP. (D) Body weight change in each group after CLP. (E) The modified SHIRPA scores on Days 1, 3, 5, and 7 following CLP. The SHIRPA score of 0–3, 4–7, and 8–12 was classified as the mild, moderate, and severe groups, respectively. All data are presented as the means ± SD. Data were analyzed using two‐way ANOVA followed by Tukey's multiple comparisons test (D), *n* = 40 in sham or *n* = 63 in CLP group.

The modified SHIRPA score has been used to define the severity grade of sepsis in mice [19, 24]. In order to provide a more intuitive representation of the health status of mice in a state of sepsis, we reduced the weight of morphological phenotypic parameters in our scoring, while increasing the importance of parameters that accurately reflect the disease state in the assessment of modified SHIRPA scores. The score included: basic vital signs, morphological appearance, skeletal reaction, gastrointestinal reaction, sensory reaction, neurological response; details see in Figure 1B. Each indicator is scored on a scale of 0–1, with 0 indicating no abnormality and 1 indicating presence, with the highest score being 12. Each mouse was marked and numbered to facilitate the documentation of score variations throughout the entire assessment process.

### 2.4 Neurological Scores

The evaluated items include corneal reflex, auricular reflex, righting reflex, avoidance reflex, and tail flick reflex. Detection of corneal reflex: by gently touching the mouse's cornea with a cotton swab, if the mouse blinks and shakes its head, then it is normal; Detection of auricular reflex: by touching the external auditory canal with a cotton swab, if it causes the mouse to avoid and turn its head vigorously, it is normal; Detection of righting reflex: Mice were placed on a platform in the supine position, and it was normal when the mice quickly turned to the prone position; Detection of avoidance reflex: the avoidance of mice is normal by temporary stimulation of the back skin of mice; Detection of tail‐flick reflex: it is normal for mice to avoid or turn away from injury by temporary stimulation of the mice tail. If mice have no reflexes, remarking as 0 point; if mice show slow reflexes (5 s < response), remarking as 1 point; if mice show sensitive reflexes (< 5 s), remarking as 2 points, and the maximum score is 10 points.

### 2.5 Neurobehavioral Tests

#### 2.5.1 Open Field Test

The open field test was performed in a box with a square open area (50 × 50 × 50 cm). The floor of the box was divided into 16 grids, of which the 4 grids in the center were defined as the central area. At the beginning of the experiment, each mouse was allowed to freely explore the box for 10 min. The average speed and time spent in the central area were recorded. Each group of mice was compared for the time spent in the central area normalized to the total time. Low center time indicates anxiety‐like behavior in the experimental mice.

#### 2.5.2 Three‐Chamber Social Test

As in the previous study, the three‐chamber sociability test was conducted in a rectangular box (40 x60 x40 cm) divided into three equal chambers [25, 26]. The experiment was evaluated in the following phases: (1) Habituation phase: Mice were released into the intermediate chamber, the left and right sliding doors were opened, and the mice were allowed to move freely between the three chambers for 10 min to familiarize themselves with the environment; (2) Sociability test: A stranger mouse (referred to as Stranger1) of the same sex as the subject mice was placed in a circular cage in one of the two chambers (left or right). The subject mice were then released into the intermediate chamber and allowed to move freely between the three chambers for 10 min. The time that the subject mice spent touching, interacting, or communicating with the unfamiliar/stranger mouse was recorded for statistical analysis. The sociability index was calculated as follows: (T(Stranger1) − T(Empty))/(T(Stranger1) + T(Empty)).

#### 2.5.3 Y Maze

Y maze trials were conducted in three long boxes (arms; 40 × 10 × 20 cm) at a 120° angle. During the test, the mouse was randomly placed in an arm and allowed to move freely from one arm to the other for 10 min. The duration of the mouse's stay in the initial and other arms was recorded. The alternation rate was calculated as: actual alternation/maximum alternation × 100%. The actual alternation value is the number of times the mouse entered the three arms in a row. The maximum alternation value is the total number of arm entries minus 2. In the Y‐maze test, a lower alternation rate indicates impaired working memory.

#### 2.5.4 Morris Water Maze Test

The Morris water maze was conducted in a circular pool (110 cm in diameter) with a constant water temperature of 20°C–22°C and a hidden circular platform (10 cm in diameter) submerged 1 cm under the water. The experiment was divided into two phases: (1) Learning phase: From Days 26 to 30 after CLP, the mice were trained to independently land on the hidden underwater platform every day. Each day, the mice were randomly placed into the water from four fixed positions. The time the mice spent to find the hidden platform was recorded (within 60 s). (2) Final test phase: Performed on Day 31 after CLP. The underwater platform was removed prior to the start of the test. The mice were gently placed in the water from one of the fixed positions. The number of times the mice crossed the platform and the time spent in the target quadrant (referred to as the northeast) were recorded.

### 2.6 Experimental Recording of Field EPSP in Isolated Brain‐LTP Recording

Animals were anesthetized and the brain isolated and placed in cooling aCSF (artificial cerebrospinal fluid) solution. After brain trimming, 350 μm thick transverse hippocampal slices were prepared using a vibrating microtome. The acute hippocampal slices were then placed in the center of an interface‐type recording chamber and incubated for at least 1 h under constant circulation with aCSF solution fluid (30°C) and 95% O_2_/5% CO_2_ carbogen supply. The stimulation electrodes Sti1 and Sti2 (stainless steel electrodes) were then placed on the Schaffer collateral fibers to study synaptic plasticity (Schaffer collateral‐to‐CA1 synapses were typically analyzed for LTP assays), and once fixed in place, the recording electrode (stainless‐steel electrode) was also placed on the Schaffer collateral branch of the CA1 region in between the stimulation electrodes [27]. A stimulus intensity that causes a maximum fEPSP (Field Excitation Potential Stimulus Response) slope of 50% was used as the test stimulus. Data points, recorded every 5 min from inputs Sti1 and Sti2, are based on the average of four field potentials evoked by biphasic constant‐current pulses (0.1 ms/polarity) at 0.2 Hz. Baseline responses (before tetanization) were recorded for at least 30 min. The slope of the fEPSPs was then measured. Synaptic strength was assessed by measuring changes in fEPSP slope relative to baseline.

### 2.7 Statistical Analysis

All results were analyzed using GraphPad Prism software (version 8.0, LaJolla, CA, USA), and the data were presented as mean ± standard deviation (mean ± SD). The Kolmogorov–Smirnov test was used to assess the Gaussian distribution of all datasets. The unpaired student's test was used to compare the two groups with Gaussian distribution data. For non‐Gaussian distribution data, Mann–Whitney U rank sum test was used for analysis. One‐way or two‐way ANOVA followed by Tukey's multiple comparisons test was used for three or more groups of data conforming to the Gaussian distribution. Non‐Gaussian distribution data were analyzed by ordinary one‐way analysis of variance (ANOVA; F‐test). Pearson and spearman correlation analyses were used to test correlations between datasets. When *p* < 0.05, the difference was considered statistically significant.

## 3 Results

### 3.1 CLP Mice Assessment by the Modified SHIRPA Score

According to the traditional methods of animal sepsis model, we performed CLP on 116 mice to induce sepsis [23, 28]. We continuously monitored health status like body weight, survival rate, and modified SHIRPA score of the mice during the 0–7 days after CLP (Figure 1A,B). Mortality curves were used to illustrate the survival rate of the mice after CLP. The survival rate in the CLP group was 54.31% (63/116, Figure 1C) within 7 days after CLP. The overall change in body weight during the 0–7 days after surgery was monitored to assess the baseline health of mice, and the CLP group experienced significant weight loss compared to the sham group (Figure 1D). The modified SHIRPA scores of all 63 surviving mice were assessed at 1, 3, 5, and 7 days post‐CLP, and the overall scoring characteristics are shown in Figure 1E. We initially used traditional methods to sort CLP mice into the three groups based on each day's score [19, 29]: a mild group with scores lower than 3, a moderate group with scores ranging from 4 to 7, and a severe group with modified SHIRPA scores higher than 8. In the cohort of surviving mice, a total of 10 CLP mice were classified as severe, while the remaining 52 mice were classified as moderate, and 1 mouse was classified as mild on Day 1 post‐CLP (Figure 1E). Notably, the modified SHIRPA scores gradually decreased as the days progressed after CLP (Figure 1E, Table 1). On Days 3 and 5 post‐CLP, a small number of mice were observed as severe/mild, whereas moderate mice predominated throughout the entire scoring period (52/63). Furthermore, on Day 7 post‐CLP, 36 mice were classified as mild, while 27 mice remained classified as moderate (Figure 1E). Nevertheless, it remains a major concern to determine whether these mice experienced SAE and to further investigate mechanisms of neuroprotection against SAE in these mice.

**TABLE 1:** Number of mice grouped by modified SHIRPA scores at each time post‐CLP.

Days post‐CLP	Number of mice in sham	Number of mice	Number of mice	Number of mice
		Mild (scores ≤ 3)	Moderate (4 ≤ scores ≤ 7)	Severe (8 ≤ scores)
1	40	1	52	10
3	40	7	51	5
5	40	17	46	0
7	40	36	27	0

### 3.2 More Than Half of the Moderate Sepsis Mice Did NOT Have Cognitive Deficits

**FIGURE 2:**
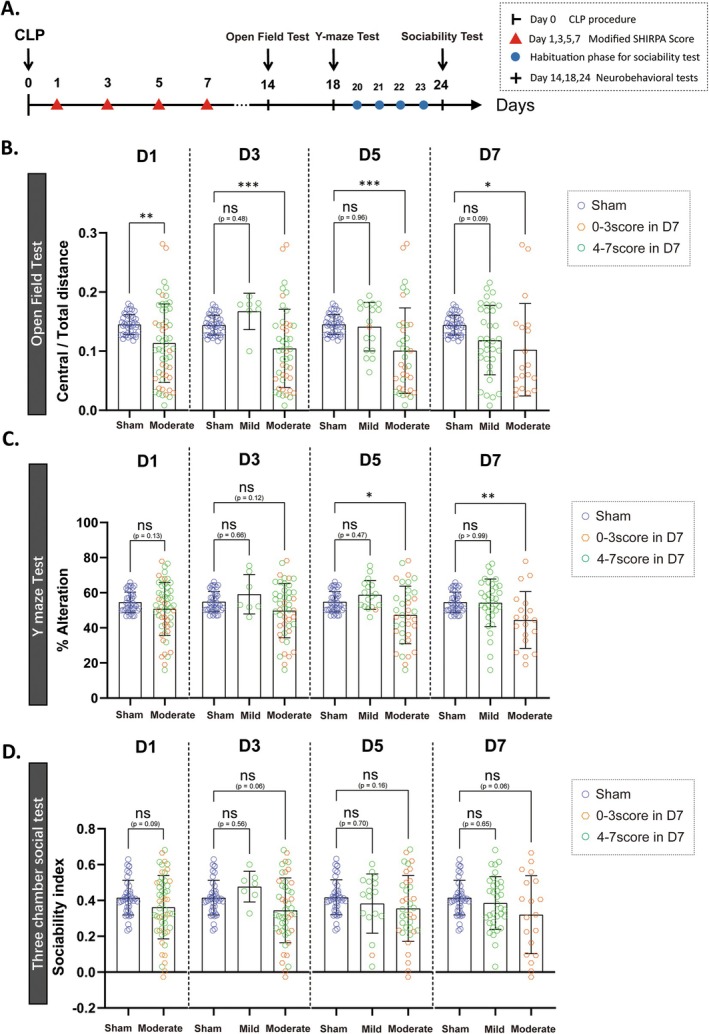
Mild and Moderate CLP mice categorized according to modified SHIRPA score do NOT demonstrate memory deficits. (A) Time course of key time points and behavioral tests. (B) The ratio of central/total distance of CLP mice in open field test 14 days post‐CLP. (C) The alteration rate in Y maze test (18 days post‐CLP). (D) The sociability index of CLP mice in three‐chamber social test (24 days post‐CLP). All data are presented as the means ± SD. Data were analyzed using the unpaired two‐tailed Student's t‐test (D1 data set) or one‐way ANOVA follow by Tukey's multiple comparisons test (D3 to D7 data set), *n* = 40 in sham and *n* = 63 in CLP group, *p < 0.05, **p < 0.01, ***p < 0.001, ns, no significance, as indicated.

A typical symptom of SAE is accompanied by long‐term memory impairment. Thus, we investigated the effect of CLP‐induced sepsis severity on cognitive function in mice by performing behavioral tests on all surviving mice during 14–24 days post‐CLP (Figure 2A). We observed that all mice belonging to the severe category (10/63 mice) and grouped by modified SHIRPA scores at Day 1 post‐CLP exhibited cognitive deficits, as evidenced by the severe mice consistently performing worse than the sham group in the open field test (Figure S1A), the Y maze test (Figure S1B), and the three‐chamber social test (Figure S1C). In addition, only the moderate mice grouped by Day 7's scores showed behavioral deficits, whereas the moderate mice (grouped by Days 1–5 scores) did not show such consistent deficits across these tests. Although the mice in the moderate group (grouped by Days 1–5 scores) demonstrated a notable increase in the distance traveled in the central area in the open field test 14 days post‐CLP (Figure 2B). However, in the Y‐maze test, the alteration rates of moderate mice (grouped by Days 1–3 scores) showed no significant differences compared to the sham group (Figure 2C). In the three‐chamber social ability test, moderate mice (grouped by Days 1–5 scores) also displayed no significant differences compared to the sham group (Figure 2D). This indicates that the moderate mice (grouped by Days 1–5 scores) showed only anxiety‐like behaviors, with no cognitive deficits in spatial memory or social abilities. Furthermore, the mice in the mild group, without exception, showed a complete lack of differences to the Sham group mice (Figure 2, green dots). Upon reviewing the scores of each mild mouse, we found that the 36 mild mice (grouped by Day 7's scores) had initially been classified in the moderate group according to the Day 1's scores, but the most of these mice did not develop cognitive impairment, meaning they are not SAE mice. Similarly, some moderate mice (grouped by 3‐day and 5‐day's scores) did not exhibit typical symptoms of SAE. This suggests that the traditional method of distinguishing SAE animals in the acute phase has shortcomings and may not accurately or effectively assess cognitive impairment in CLP mice with encephalopathy. Therefore, a more appropriate method of identifying SAE animals, particularly in the acute phase of CLP, needs to be established.

### 3.3 A Novel Median‐Division Method for Grouping Modified SHIRPA Scores Reveals Cognitive Deficits in CLP Mice

**FIGURE 3:**
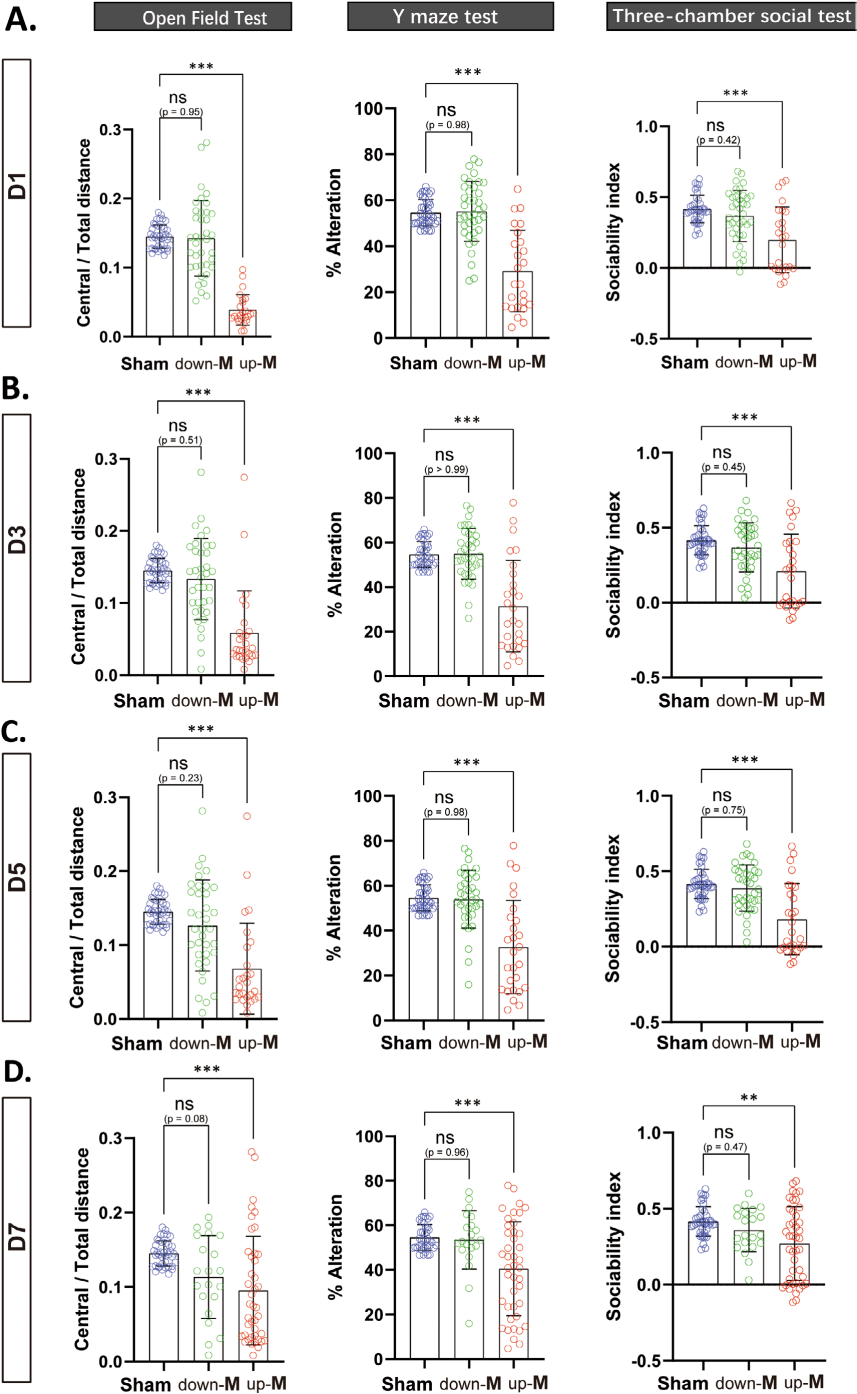
The new division method of the median of modified SHIRPA score demonstrated cognitive deficits in Up‐M group mice. The behavioral statistics (among open field test, Y maze test and three‐chamber social test were performed on Days 14, 18, and 24, respectively) in Sham, up‐M group, and down‐M group, defined by the median division on Day 1 (A), Day 3 (B), Day 5 (C), Day 7 (D) after CLP, respectively. All data are presented as the means ± SD. Data were analyzed using one‐way ANOVA followed by Tukey's multiple comparisons test, *n* = 40 in sham or *n* = 63 in CLP group, *p < 0.05, **p < 0.01, ***p < 0.001, ns, no significance, as indicated.

To explore a more effective and accurate method for assessing cognitive deficits in CLP mice, we hypothesize a novel median division approach to categorize the severity of septic mice. By calculating the median values of the scores of all CLP mice on each day, we used the median to dynamically divide the CLP mice into two subgroups—the up‐M mice/group and down‐M mice/group. The median scores at each time point for the CLP mice are shown in Figure S2, with the median of Day 1 at 7 (*M*_D1_ = 7), Day 3 at 6 (*M*_D3_ = 6), Day 5 at 5 (*M*_D5_ = 5) and Day 7 at 3 (*M*_D7_ = 3). The median divided the mice into two groups: the up‐median group (up‐M) and the down‐median group (down‐M). We subsequently analyzed the results of all behavioral tests (on Days 14–24 post‐CLP) performed on CLP mice between these two groups. On Day 1 post‐CLP, down‐M mice showed no significant differences in the three behavioral tests, whereas up‐M mice demonstrated significantly poorer performance compared to the Sham group (Figure 3A). The grouping of down‐M mice (grouped by Days 3, 5, and 7, respectively) consistently showed no significant differences across all behavioral tests relative to the sham group, whereas up‐M mice again performed significantly worse (Figure 3B–D). This suggested that the new method of grouping using the median division of the modified SHIRPA scores system was more effective in identifying SAE mice.

### 3.4 Validation of the Median‐Division Method Using the ROC Curves and the Neurological Scores

**FIGURE 4:**
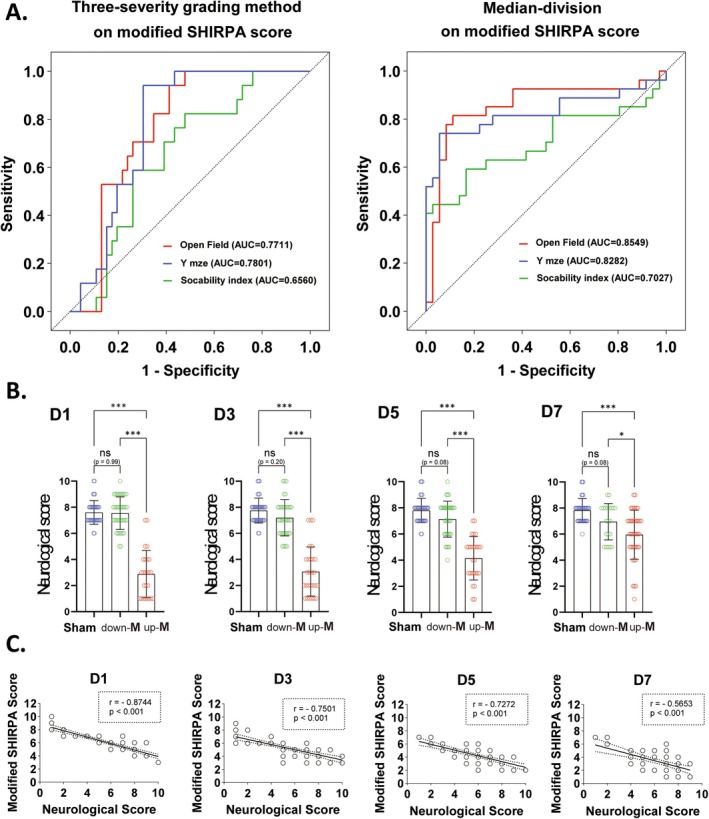
ROC curves and the neurological scores from the median division of the modified SHIRPA score validated its effectiveness in identifying neurological deficits in CLP mice. (A) (The left figure) When employing three‐severity grading method in the modified SHIRPA score, curve in red represents the performance in the open filed test (AUC = 0.7711), curve in blue represents the performance in the Y‐maze test (AUC = 0.7801), and curve in blue represents the performance in the three‐chamber social tests (AUC = 0.6560). (The right figure) When employing median‐division method in the modified SHIRPA score, curve in red represents the performance in the open filed test (AUC = 0.8549), curve in blue represents the performance in the Y‐maze test (AUC = 0.8282), and curve in blue represents the performance in the three‐chamber social tests (AUC = 0.7027). (B) The neurological scores of mice in different groups (sham, down‐M, and up‐M) from Day 1 to Day 7 post‐CLP, *n* = 40 in sham or *n* = 63 in CLP group. (C) Spearman correlation analysis of the modified SHIRPA score with the neurological scores at different Days 1, 3, 5 and 7 post‐CLP, respectively. *r*: Spearman correlation coefficient, *n* = 40 in sham or *n* = 63 in CLP group. All data are expressed as the means ± SD. Data were analyzed using the one‐way ANOVA followed by Tukey's multiple comparisons test, *p < 0.05, **p < 0.01, ***p < 0.001, ns, no significance, as indicated.

To intuitively validate the feasibility of using the median‐division method to identify CLP mice with cognitive deficits, we first used ROC curves of the modified SHIRPA score in relation to the primary results of behavioral tests to validate the predictive performance of the median division method. When employing the mild–moderate–severe categorization, the area under the curve (AUC) for the open field test was 0.77, for the Y‐maze it was 0.78, and for the sociability test it was 0.66 (Figure 4A). In contrast, when using the median‐division method, the AUCs for the open field test, Y‐maze, and sociability test were 0.85, 0.83, and 0.70, respectively (Figure 4A). The median‐division method significantly enhanced the AUC values of the ROC curves, indicating its superior predictive performance for identifying cognitive deficits. Next, we scored all mice using a neurological scoring system that can determine neurological impairment. The results showed that the neurological scores of down‐M mice consistently did not exhibit any differences compared with the sham group 1–7 days post‐CLP, while the neurological scores of up‐M mice were significantly lower than the down‐M group and the sham group, respectively (Figure 4B). These data suggested that the neurological score aligned with the grouping criteria based on the median of the modified SHIRPA score, thereby confirming the feasibility of the median division method from the perspective of the nervous system. Notably, during the correlation analysis of the median‐division systems, we observed that both median‐division systems displayed a strong negative correlation due to their inversely proportional scoring characteristics (Figure 4C). These data indicate that the median‐division method of the modified SHIRPA score does indeed identify neurological impairments in CLP mice already a few days after CLP.

### 3.5 Independent Ability of Median Division to Predict Cognitive Deficits in CLP Mice

To evaluate the efficacy of the median‐division method for identifying cognitive deficits in a murine sepsis model, we utilized an additional cohort of CLP mice (*n* = 50) to validate our novel approach for identifying the SAE. The survival rate in this cohort was 54%. The body weight, survival rate curve, and modified SHIRPA score of this group are presented in Figure S3. Following the same procedure, the mice were divided into the up‐M and the down‐M groups according to their different time points post‐CLP. In the open field test, up‐M mice (grouped by all the time points) traveled a significantly shorter distance in the central area compared to the sham group, while down‐M mice displayed no significant differences on Day 14 post‐CLP (Figure S4A). Similarly, in the Y‐maze test and the three‐chamber social test, the up‐M mice have significantly shown cognitive impairment compared to the sham group, whereas down‐M mice did not exhibit any differences on Day 18 and Day 22 post‐CLP (Figure S4B,C). Taken together, these results collectively indicated that the application of the modified SHIRPA scoring system, particularly when employing the median division method, serves as an effective tool for identifying CLP mice with cognitive deficits.

### 3.6 CLP Induced Neuronal Death and Microglial Polarization in the Hippocampus and Cortex of the Up‐M Mice, but Not in the Down‐M Mice

**FIGURE 5:**
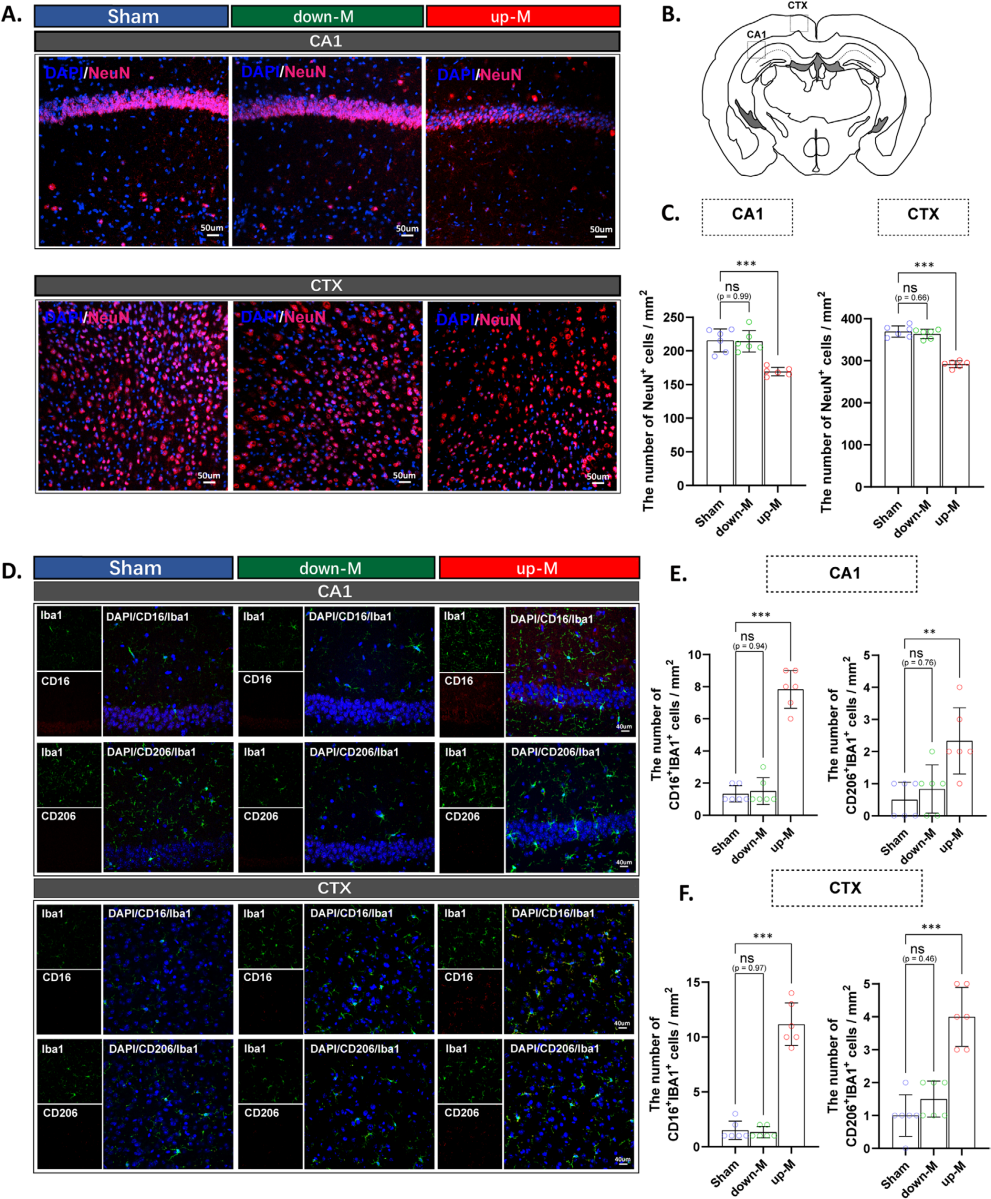
CLP induced neuronal death and microglia polarization in the region of the hippocampus and cortex of up‐M mice, but not in down‐M mice. (A) Representative fluorescent images of NeuN immunostaining and DAPI in the hippocampus CA1 (CA1) and cortex (CTX) area (scale bar = 50 μm). (B) Schematic representation of the CTX and CA1 regions in mouse brain slices. (C) Quantification of NeuN‐positive cells in the CA1 and CTX area. (D) Representative fluorescence images of CD16^+^Iba1^+^ and CD206^+^Iba1^+^ immunostaining in the CA1 and CTX area (scale bar = 40 μm). (E) Quantification of CD16^+^Iba1^+^ and CD206^+^Iba1^+^ cells in the CA1 area. (F) Quantification of CD16^+^Iba1^+^ and CD206^+^Iba1^+^ cells in the CTX area. All data are presented as the means ± SD. Data were analyzed using the one‐way ANOVA followed by Tukey's multiple comparisons test. *n* = 6/group, *p < 0.05, **p < 0.01, ***p < 0.001, ns: No significance, as indicated.

Neuronal damage and neuroinflammation are typical pathological injury characteristics of SAE [30]. We next used immunofluorescent staining of the neuronal nuclear marker NeuN to assess the extent of brain tissue damage in the hippocampus CA1 and cerebral cortex (CTX) of CLP mice at 24 days post‐CLP (Figure 5A,B). The results revealed that the up‐M mice had a significant reduction in the number of neurons in both the CA1 and CTX compared to the sham group, whereas the down‐M group showed no significant differences, indicating a significant neuronal death in the brain of up‐M mice (Figure 5C). Subsequently, we performed immunofluorescence staining for microglia and their pro‐inflammatory activation marker CD16, as well as the anti‐inflammatory activation marker CD206, to assess microglial activation in the hippocampus CA1 and CTX of CLP mice (Figure 5D). The results revealed that, compared to the sham group, the up‐M mice exhibited a significant increase in the number of pro‐inflammatory microglia (CD16^+^Iba1^+^) and anti‐inflammatory microglia (CD206^+^Iba1^+^) in both the CA1 (Figure 5E) and CTX (Figure 5F), while the down‐M group showed no significant differences, indicating the presence of microglial activation in the brain of the up‐M mice. This indicates that the median division of the mice into the up‐M group resulted in a homogeneous group of mice with significantly greater neuronal death and microglial polarization in the hippocampus CA1 and CTX.

### 3.7 Impaired Spatial Learning and Memory Ability and Hippocampal LTP in Up‐M Mice, but not in Down‐M Mice

**FIGURE 6:**
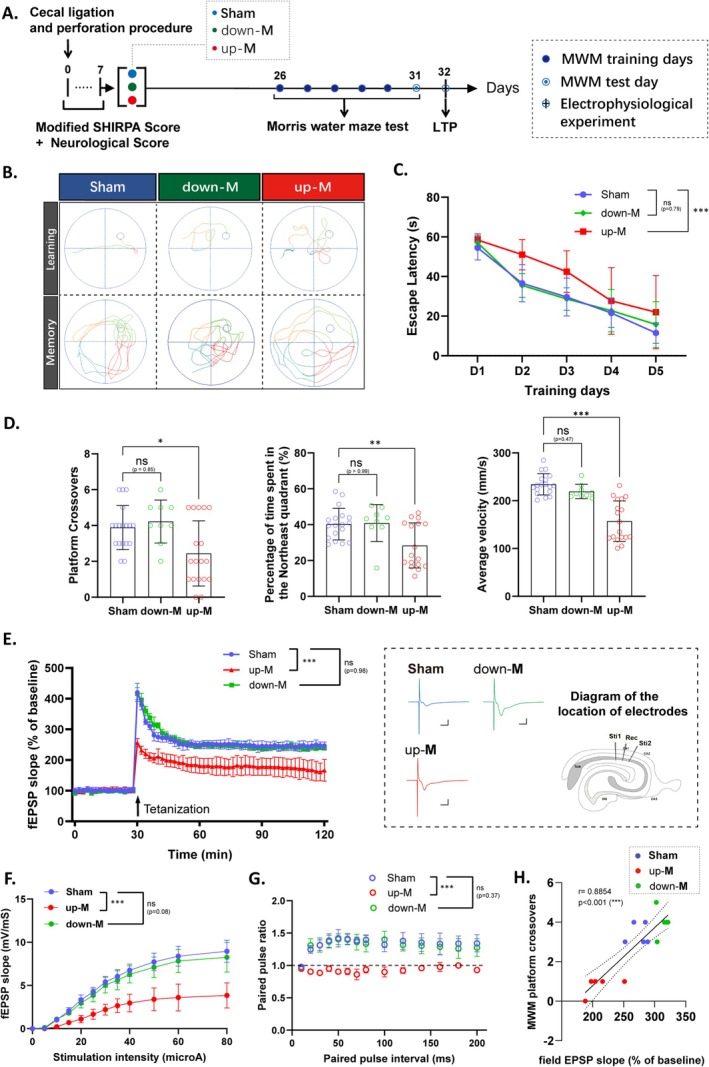
Impaired spatial learning/memory ability and LTP of hippocampus in the up‐M mice, but not in down‐M mice. (A) Schematic timeline of animal handling procedures concluding in the Morris water maze tests and electrophysiological experiments. (B) Representative swimming paths of the three groups during the training and memory test phases of the Morris water maze test. (C) The escape latency in the learning days, *n* = 18 in sham and *n* = 27 in CLP group. (D) The number of platform crossovers and the percentage of time spent in the target quadrant and the average speed of mice in the memory phase, *n* = 18 in sham or *n* = 27 in CLP group. (E) LTP was recorded on transversal hippocampal slices from different groups (Sham, down‐M, up‐M, *n* = 5/group). Time course of fEPSP recorded and plotted for every data point in the hippocampal CA1 region based on the average of four field potentials evoked by biphasic constant‐current pulses (0.1 ms/polarity) at 0.2 Hz in the Schaffer collateral region. The vertical arrow indicates the time point of tetanization. The colored example traces and the diagram of the location of electrodes matched with the LTP fEPSP scatter graph. (F) The input/output functions for stimulus intensity versus fEPSP slope for the different groups of CLP mice are presented (Sham, down‐M, up‐M, *n* = 5/group). (G) The paired pulse ratio for the CLP mice during the LTP procedure (Sham, down‐M, up‐M, *n* = 5/group). (H) Spearman correlation analysis between the Morris water maze main indicator and the field EPSP slope of LTP (Sham, down‐M, up‐M, *n* = 5/group). All data are presented as the means ± SD. Data were analyzed using the one‐way or two‐way ANOVA followed by Tukey's multiple comparisons test. *p < 0.05, **p < 0.01, ***p < 0.001, ns, no significance, as indicated.

Sepsis survivors have been shown to have long‐term impairments in learning and memory [9]. To further validate the ability of the median division to identify CLP mice exhibiting SAE, we assessed LTP (Day 32 post‐CLP) following the Morris water maze test (Days 26–31 post‐CLP) to provide a more intuitive observation of which group of mice experienced learning and memory deficits (Figure 6A). The results indicate that, compared to the sham group, the up‐M group exhibited significant impairments in spatial memory and learning compared to the sham group, as evidenced by a significantly prolonged escape latency (Figure 6C), a reduction in the number of platform crossings, a decreased average speed, and a reduced percentage of time spent in the target quadrant (Figure 6D). In contrast, the down‐M group showed no significant differences when compared to the sham group. These results indicate that both learning and spatial memory are impaired in up‐M mice. Next, we measured the LTP induced by high‐frequency stimulation of the Schaffer collateral pathway. As shown in Figure 6E, the up‐M mice showed reduced LTP compared to the sham group, while the down‐M mice showed no significant differences (Figure 6E). We also examined paired pulse stimulation and the dependence of field potential size on different stimulation intensities to characterize basal synaptic transmission in these groups. Brain slices from up‐M mice showed a significantly lower increase in field potential at different stimulation intensities compared to the other two groups (Figure 6F). In addition, the paired pulse ratio of up‐M mice was significantly lower than that of sham and down‐M mice (Figure 6G). Analyzing the correlation between fEPSP (slope % of baseline) 10 min after LTP stimulation and the main index of the mice in the Morris water maze (number of platform crossings in the final probe test), we found a strong correlation (Figure 6H). Thus, the electrophysiological experiments support the impairment of LTP and synaptic plasticity in the hippocampal region of up‐M mice. Taken together, our results suggest that the up‐M mice, classified by median division, have significant deficits in learning and memory, which are associated with reduced LTP and impaired synaptic plasticity in the hippocampus.

## 4 Discussion

Despite numerous early warning models and continuous updating of cluster management strategies, severe sepsis remains associated with high morbidity and mortality rates [31]. SAE is often comorbid in these patients, complicating differential diagnosis and treatment, and worsening the prognosis [32]. Research shows that the incidence of SAE in sepsis patients varies widely, from 8% to 65%, depending on the severity of sepsis, patient characteristics, and diagnostic criteria [33]. In this study, we used a moderate CLP protocol based on the method established by Daniel Rittirsch et al. [23]. Approximately half of the mice developed neurological deficits, aligning with the average incidence of SAE reported in clinical settings. Similar to the clinical challenges in defining SAE, there is a lack of clear, standardized diagnostic criteria for SAE in animal models.

The overall physiological status of septic mice, including neurological function, is commonly assessed using the modified SHIRPA score [34]. Scores are categorized as mild (0–3), moderate (4–7), and severe (8–12). Our results showed that most CLP mice fell into the moderate category, although their disease phenotypes varied considerably. Mice with severe scores displayed the most pronounced symptoms but often succumbed early in sepsis. We then conducted behavioral tests to evaluate cognitive deficits in CLP mice. In both the Y maze test, assessing motor function and spatial memory, and the three‐chamber social test, evaluating social interaction and novelty recognition, moderate mice did not show significantly worse cognitive performance. However, they exhibited pronounced anxiety‐like behaviors in the open field test. This suggests that not all moderately injured mice develop SAE.

In this study, we used the median‐derived modified SHIRPA score as a screening index for SAE. Mice with scores above the median showed significant neurological impairment in all neurobehavioral tests compared to controls. We further evaluated the encephalopathic features of septic mice using the neuroreflex score criteria, which revealed statistically significant differences between groups. This supports the feasibility of using this screening method to identify SAE in mice.

To further validate this approach, we conducted a second cohort study using behavioral, histological, and electrophysiological assessments. The results showed that the up‐M group exhibited worse learning and memory across all behavioral tests, including the open field test (Day 14 post‐CLP), Y‐maze test (Day 18), sociability test (Day 24), and Morris water maze test (1 month). Evidence suggests that cognitive deficits in CLP mice are linked to neuronal death and acute‐phase neuroinflammation [22, 35]. We observed a significant reduction in neuron numbers and notable microglial activation in the cerebral cortex and hippocampal CA1 region of up‐M mice. It was noted that higher modified SHIRPA scores correlated with more severe neuropathological damage. However, this association requires further validation. Synaptic dysfunction, including loss of plasticity and synapse degeneration, is closely linked to cognitive decline [36]. In our study, the up‐M group showed reduced LTP levels and impaired synaptic efficacy in electrophysiological tests (Day 32 post‐CLP). In conclusion, the median screening method using the modified SHIRPA score effectively discriminated between Sham and CLP mice with postoperative encephalopathy mice and also differentiated non‐encephalopathic from encephalopathic mice after CLP surgery.

SAE is characterized by diffuse brain dysfunction [6]. Evidence shows that both sepsis and SAE are highly heterogeneous, presenting with diverse neuropathological impairments [37, 38]. While animal models of sepsis do not fully replicate human disease or the clinical care provided to sepsis patients, there is potential to enhance these models by better staging of sepsis. Improved methods for categorizing and staging SAE in animal models are also needed. This would enhance the comparability of studies and help resolve conflicting results across different models. Given the complexity of SAE, a multi‐target therapeutic approach might be necessary. The current challenge may lie in accurately identifying true SAE cases. Enhanced differentiation of SAE in animal models could aid in identifying target populations for more precise and cost‐effective clinical trials. Additionally, we anticipate that improved recovery from encephalopathy will positively impact overall prognosis, including restoring central nervous system regulation of immune and endocrine functions, thereby supporting faster patient recovery beyond neurological function alone.

Nevertheless, this study has several limitations. For instance, we used only the CLP method to establish the septic mouse model. The applicability of the median‐derived modified SHIRPA score to screening sepsis models induced by LPS or other methods remains to be validated. In this research, we evaluated neurological impairment in mice 1 month after surgery. However, long‐term outcomes remain uncertain, such as whether SAE mice develop more pronounced brain atrophy at 6 months post‐surgery.

In summary, we used the CLP method to create a mouse model of sepsis. We hypothesized that sepsis occurs in mice with clinical scores above the median modified SHIRPA score, consistent with clinical observations that SAE occurs in more than half of sepsis cases. This hypothesis was confirmed by assessments of neurological function, behavioral tests, and neurophysiological measurements. Finally, we have established a standardized assessment method for SAE in animal models, providing a consistent framework for future SAE research. By providing a robust and reproducible framework for evaluating SAE in the mouse models, our findings may pave the way for the development of more precise diagnostic criteria in clinical work. This could enable researchers to identify SAE earlier and more accurately, facilitating timely interventions that could mitigate long‐term neurological impairments in patients.

## 5 Conclusion

Our study establishes a mouse model of SAE and demonstrates that the median‐division grouping method of the modified SHIRPA score effectively discriminates the severity of sepsis and predicts cognitive impairment. This approach is straightforward and reliable, facilitating future research on the mechanisms of cognitive dysfunction related to sepsis.

## Conflicts of Interest

The authors declare no conflicts of interest.

## Supporting information


Appendix S1:

## Data Availability

Data sharing is not applicable to this article as no datasets were generated. Scripts for data analysis are available from the corresponding author upon reasonable request.
