# Supplementary material for: Optimized Mouse Model of Sepsis‐Associated Encephalopathy: A Rational Standard Based on Modified SHIRPA Score and Neurobehaviors in Mice
Source: CNS Neurosci Ther. 2025 Apr 9;31(4):e70365. doi: 10.1111/cns.70365 (PMC11979712; doi:10.1111/cns.70365)
Supplement: Supplementary file 1 — Appendix S1: [file CNS-31-e70365-s001.pdf]

Supplementary Materials

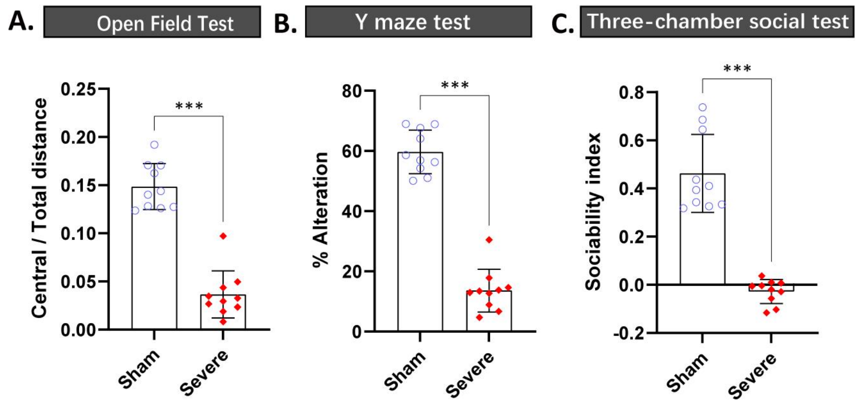

Figure S1. Severe CLP mice with SHIRPA score always demonstrate memory deficits. A. The ratio of central distance of CLP mice in open field test 14 days post-CLP. **B.** The alteration rate in Y-maze test (18 days post-CLP). **C.** The sociability index of CLP mice in three-chamber social test (24 days post-CLP). All data are presented as the means±SEM and analyzed using the unpaired two tails Student's t-test, n=10/group, ***p<0.001, ns: no significance, as indicated.

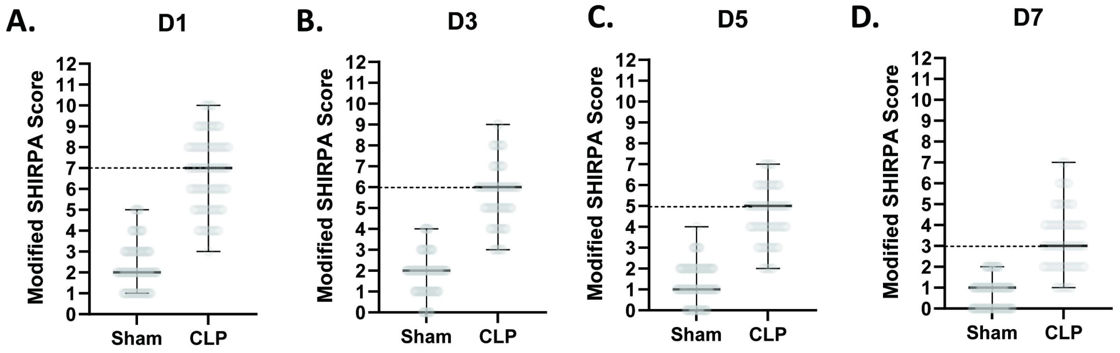

Figure S2. The modified SHIRPA score and the median scores at each postoperative time point for the CLP mice. A. The overall median score on day 1 after CLP. **B.** The overall median score on day 3 after CLP. **C.** The overall median score on day 5 after CLP. **D.** The overall median score on day 7 after CLP. n=40 in sham or n=63 in CLP group.

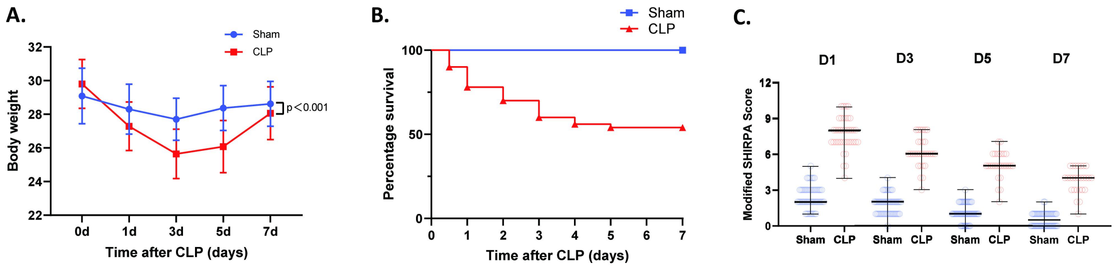

Figure S3. Modified SHIRPA Score, body weight, and the survival rate of second group of CLP mice from testing the Median-division method. A Body weight change in each group after CLP. **B** The survival rate 7 days after CLP. **C** The modified SHIRPA scores on day 1, 3, 5, and 7 following CLP. All data are presented as the means±SD and analyzed using the two way ANOVA, n=18 in sham or n=27 in CLP group, **p*<0.05, ***p*<0.01, ****p*<0.001, ns: no significance, as indicated.

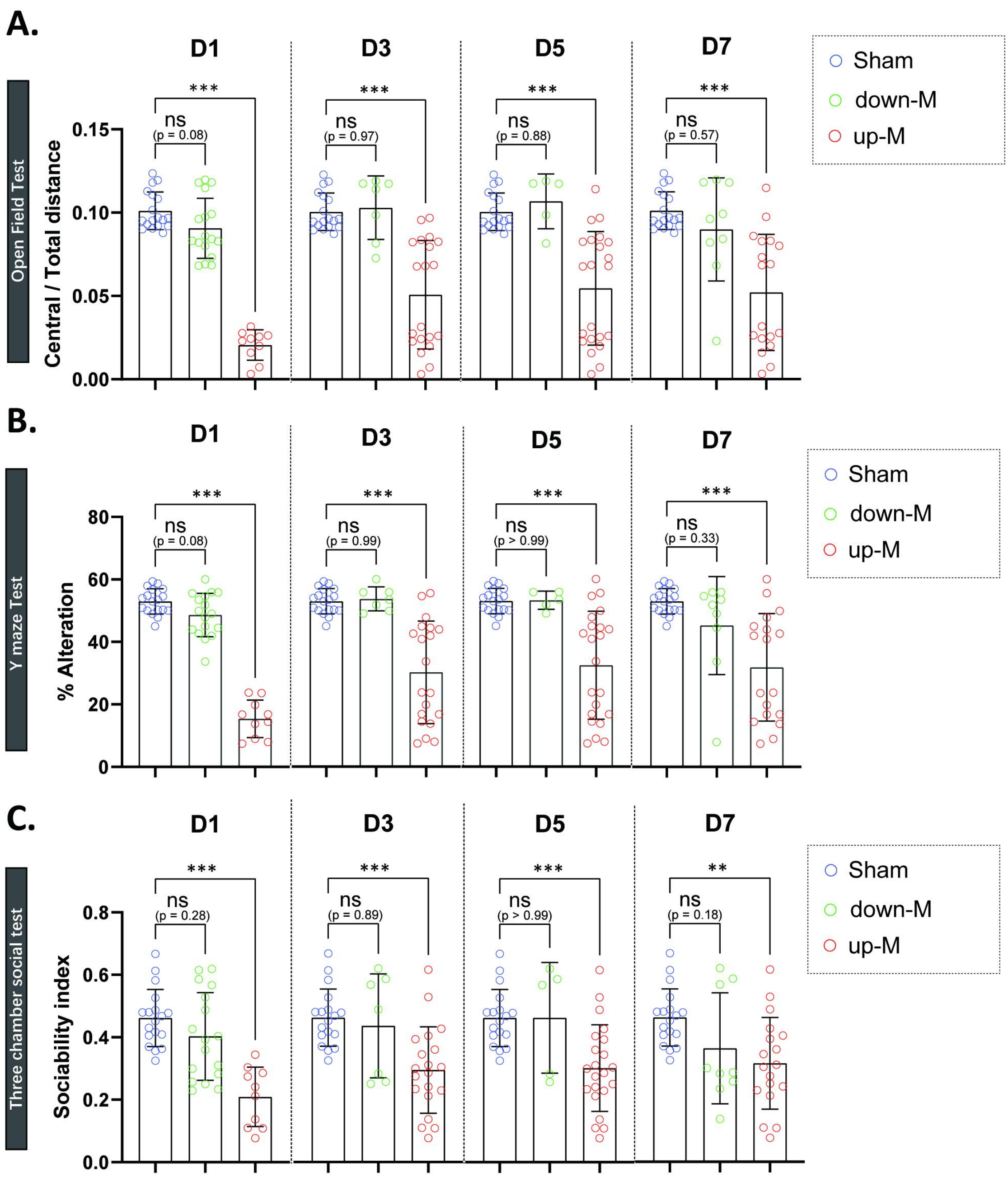

Figure S4. Another batch of CLP mice comfirmed the accuracy of the median method in identifying SAEs. A. The ratio of central/total distance of CLP mice in open field test 14 days post-CLP. **B** The alteration rate in Y maze test (18 days post-CLP). **C** The sociability index of CLP mice in three-chamber social test (24 days post-CLP). All data are expressed as the means±SD. Date were analyzed using one-way ANOVA followed by Tukey's multiple comparisons test. n=18 in sham or n=27 in CLP group, **p*<0.05, ***p*<0.01, ****p*<0.001, ns: no

significance, as indicated.

Table S1. The characteristics of the modified SHIRPA score

D1

Groups		n	Modified SHIRPA score													mean±SD
			0	1	2	3	4	5	6	7	8	9	10	11	12	
Sham		40	0	9	18	10	3	0	0	0	0	0	0	0	0	2.18±0.87
CLP	Mild	1	0	0	0	1	0	0	0	0	0	0	0	0	0	/
	Moderate	52	0	0	0	0	11	13	13	15	0	0	0	0	0	5.62±1.12
	Severe	10	0	0	0	0	0	0	0	0	6	3	1	0	0	8.50±0.71

D3

Groups		n	Modified SHIRPA score													mean±SD
			0	1	2	3	4	5	6	7	8	9	10	11	12	
Sham		40	1	9	25	3	2	0	0	0	0	0	0	0	0	1.90±0.78
CLP	Mild	7	0	0	0	7	0	0	0	0	0	0	0	0	0	/
	Moderate	51	0	0	0	0	11	18	20	2	0	0	0	0	0	5.25±0.84
	Severe	5	0	0	0	0	0	0	0	0	4	1	0	0	0	8.20±0.45

D5

Groups		n	Modified SHIRPA score													mean±SD
			0	1	2	3	4	5	6	7	8	9	10	11	12	
Sham		40	2	16	20	2	0	0	0	0	0	0	0	0	0	1.55±0.68
CLP	Mild	17	0	0	6	11	0	0	0	0	0	0	0	0	0	2.65±0.49
	Moderate	46	0	0	0	0	19	18	6	3	0	0	0	0	0	4.85±0.89
	Severe	0	0	0	0	0	0	0	0	0	0	0	0	0	0	/

D7

Groups		n	Modified SHIRPA score													mean±SD
			0	1	2	3	4	5	6	7	8	9	10	11	12	
Sham		40	14	22	4	0	0	0	0	0	0	0	0	0	0	0.75±0.63
CLP	Mild	36	0	6	15	15	0	0	0	0	0	0	0	0	0	2.25±0.73
	Moderate	27	0	0	0	0	14	7	4	2	0	0	0	0	0	4.78±0.97
	Severe	0	0	0	0	0	0	0	0	0	0	0	0	0	0	/

Table S2.The primary results of behavioral tests of CLP mice grouped by three-severity grading method

Open Field Test

	D1	D3	D5	D7
Sham	0.15±0.02	0.15±0.02	0.15±0.02	0.15±0.02
Severe	0.04±0.02 (***p<0.001)	0.04±0.03 (***p<0.001)	/	/
Moderate	0.11±0.07 (**p=0.004)	0.1±0.07 (***p<0.001)	0.1±0.07 (***p<0.001)	0.1±0.08 (*p=0.01)
Mild	/	0.17±0.03 (ns,p=0.48)	0.14±0.04 (ns,p=0.96)	0.12±0.06 (ns,p=0.09)

Note:*p<0.05, **p<0.01, ***p<0.001, ns: no significance, compared with sham group

Y maze Test

	D1	D3	D5	D7
Sham	54.61±5.77	54.61±5.77	54.61±5.77	54.61±5.77
Severe	13.61±7.11 (***p<0.001)	13.61±7.11 (***p<0.001)	/	/
Moderate	50.76±15.14 (ns,p=0.13)	45.82±18.56 (ns,p=0.12)	47.1±16.34 (*p=0.01)	44.47±16.14 (**p=0.006)
Mild	/	58.84±11.19 (ns,p=0.66)	58.51±8.21 (ns,p=0.47)	53.24±14.06 (ns,p>0.99)

Note:*p<0.05, **p<0.01, ***p<0.001, ns: no significance, compared with sham group

Three-chamber social test

	D1	D3	D5	D7
Sham	0.42±0.1	0.42±0.1	0.42±0.1	0.42±0.1
Severe	-0.03±0.05 (***p<0.001)	-0.03±0.05 (***p<0.001)	/	/
Moderate	0.36±0.18 (ns,p=0.99)	0.33±0.2 (ns,p=0.06)	0.35±0.18 (ns,p=0.16)	0.32±0.22 (ns,p=0.06)
Mild	/	0.48±0.09 (ns,p=0.56)	0.38±0.16 (ns,p=0.70)	0.39±0.15 (ns,p=0.65)

Note:*p<0.05, **p<0.01, ***p<0.001, ns: no significance, compared with sham group

Table S3.The primary results of behavioral tests of CLP mice grouped by median-division method

Open Field Test

	D1	D3	D5	D7
Sham	0.15±0.02	0.15±0.02	0.15±0.02	0.15±0.02
down-M	0.14±0.05 (ns,p=0.95)	0.13±0.06 (ns,p=0.51)	0.13±0.06 (ns,p=0.23)	0.11±0.06 (ns,p=0.08)
up-M	0.03±0.02 (***p<0.001)	0.06±0.06 (***p<0.001)	0.07±0.06 (***p<0.001)	0.1±0.07 (***p<0.001)

Note:*p<0.05, **p<0.01, ***p<0.001, ns: no significance, compared with sham group

Y maze Test

	D1	D3	D5	D7
Sham	54.61±5.77	54.61±5.77	54.61±5.77	54.61±5.77
down-M	55.15±13.06 (ns,p=0.98)	54.92±11.36 (ns,p>0.99)	53.98±12.89 (ns,p=0.96)	53.51±13.15 (ns,p=0.96)
up-M	29.23±17.74 (***p<0.001)	31.46±20.55 (***p<0.001)	32.72±20.76 (***p<0.001)	40.54±21.05 (***p<0.001)

Note:*p<0.05, **p<0.01, ***p<0.001, ns: no significance, compared with sham group

Three-chamber social test

	D1	D3	D5	D7
Sham	0.42±0.1	0.42±0.1	0.42±0.1	0.42±0.1
down-M	0.37±0.18 (ns,p=0.42)	0.37±0.16 (ns,p=0.45)	0.39±0.15 (ns,p=0.75)	0.36±0.14 (ns,p=0.47)
up-M	0.2±0.23 (***p<0.001)	0.21±0.25 (***p<0.001)	0.18±0.24 (***P<0.001)	0.27±0.24 (**p=0.001)

Note:*p<0.05, **p<0.01, ***p<0.001, ns: no significance, compared with sham group

Table S4. The characteristics of the neurological score

D1

Groups		n	Neurological score											mean±SD
			0	1	2	3	4	5	6	7	8	9	10	
Sham		40	0	0	0	0	0	0	2	19	14	3	2	7.6±0.9
CLP	down-M	38	0	0	0	0	0	2	6	10	10	9	1	7.55±1.22 (ns,p=0.99)
	up-M	25	0	8	3	6	4	2	0	2	0	0	0	2.88±1.73 (***p<0.001)

Note:*p<0.05, **p<0.01, ***p<0.001, ns: no significance, compared with sham group

D3

Groups		n	Neurological score											mean±SD
			0	1	2	3	4	5	6	7	8	9	10	
Sham		40	0	0	0	0	0	0	2	15	17	3	3	7.75±0.95
CLP	down-M	36	0	0	0	0	0	4	8	9	10	2	3	7.19±1.37 (ns,p=0.20)
	up-M	27	0	6	7	4	5	2	0	3	0	0	0	3.07±1.84 (***p<0.001)

Note:*p<0.05, **p<0.01, ***p<0.001, ns: no significance, compared with sham group

D5

Groups		n	Neurological score											mean±SD
			0	1	2	3	4	5	6	7	8	9	10	
Sham		40	0	0	0	0	0	0	2	13	19	3	3	7.8±0.94
CLP	down-M	36	0	0	0	0	1	4	7	6	14	3	1	7.14±1.34 (ns,p=0.08)
	up-M	27	0	2	2	7	2	9	3	2	0	0	0	4.15±1.61 (***p<0.001)

Note:*p<0.05, **p<0.01, ***p<0.001, ns: no significance, compared with sham group

D7

Groups		n	Neurological score											mean±SD
			0	1	2	3	4	5	6	7	8	9	10	
Sham		40	0	0	0	0	0	0	1	14	19	3	3	7.83±0.9
CLP	down-M	21	0	0	0	0	0	5	3	3	8	2	0	6.95±1.36 (ns,p=0.08)
	up-M	42	0	1	3	0	3	8	8	11	6	2	0	5.52±1.91 (***p<0.001)

Note:*p<0.05, **p<0.01, ***p<0.001, ns: no significance, compared with sham group

Table S5.Quantification of immunofluorescent staining

The number of NeuN$^+$ cells / mm^2 in hippocampus CA1

Sham	192	225	231	198	215	232	215.5±17.1
down-M	205	211	243	221	198	207	214.17±16.03 (ns,p=0.99)
up-M	179	165	170	161	168	173	169.33±6.28 (***p<0.001)

Note:*p<0.05, **p<0.01, ***p<0.001, ns: no significance, compared with sham group

The number of NeuN$^+$ cells / mm^2 in cerebral cortex

Sham	378	354	389	357	364	375	369.5±13.49
down-M	352	372	368	348	374	369	363.83±11 (ns,p=0.66)
up-M	295	301	287	296	278	293	291.67±8.09 (***p<0.001)

Note:*p<0.05, **p<0.01, ***p<0.001, ns: no significance, compared with sham group

The number of CD16$^+$IBA1$^+$ cells / mm^2 in hippocampus CA1

Sham	1	2	1	1	1	2	1.33±0.52
down-M	1	1	2	1	3	1	1.5±0.84 (ns,p=0.94)
up-M	8	7	9	8	6	9	7.83±1.17 (***p<0.001)

Note:*p<0.05, **p<0.01, ***p<0.001, ns: no significance, compared with sham group

The number of CD206$^+$IBA1$^+$ cells / mm^2 in hippocampus CA1

Sham	1	0	1	0	0	1	0.5±0.55
down-M	1	0	1	0	2	1	0.83±0.75 (ns,p=0.76)
up-M	3	2	2	4	1	2	2.33±1.03 (**p=0.003)

Note:*p<0.05, **p<0.01, ***p<0.001, ns: no significance, compared with sham group

The number of CD16$^+$IBA1$^+$ cells / mm^2 in cerebral cortex

Sham	1	2	1	3	1	1	1.5±0.84
down-M	1	2	1	1	1	2	1.33±0.52 (ns,p=0.97)
up-M	10	11	13	10	9	14	11.17±1.94 (***p<0.001)

Note:*p<0.05, **p<0.01, ***p<0.001, ns: no significance, compared with sham group

The number of CD206$^+$IBA1$^+$ cells / mm^2 in cerebral cortex

Sham	1	0	1	2	1	1	1±0.63
down-M	1	2	1	2	2	1	1.5±0.55 (ns,p=0.46)
up-M	4	5	4	3	5	3	4±0.89 (***p<0.001)

Note:*p<0.05, **p<0.01, ***p<0.001, ns: no significance, compared with sham group

Table S6. Statistical analysis of the primary results from the Morris water maze

Escape latency in Morris water maze

	D1	D2	D3	D4	D5
Sham	54.48±5.94	36.6±9.11	29.64±9.25	21.53±7.05	11.49±5.02
down-M	57.03±2.93	35.49±5.66	28.61±5.37	22.79±10.09	15.78±10.86
up-M	58.46±2.98	51±7.4	42.45±10.22	27.67±16.38	22±17.99

Tukey's multiple comparisons test	Predicted (LS) mean diff.	95.00% CI of diff.	Summary	Adjusted P Value
Sham vs. down-M	-1.19	-5.530 to 3.150	ns	0.79
Sham vs. up-M	-9.567	-13.11 to -6.024	***	<0.001

Memory abilities in Morris water maze

	Platform Crossovers	Time spent (%) in the Northeast quadrant	Average velocity (mm/s)
Sham	3.89±1.23	40.3±8.81	234.35±22.28
down-M	4.22±1.2 (ns,p=0.85)	40.88±10.31 (ns,p>0.99)	219.38±14.89 (ns,p=0.47)
up-M	2.44±1.82 (*p=0.02)	28.38±12.56 (**p=0.006)	157.32±42.39 (***p<0.001)

Note:*p<0.05, **p<0.01, ***p<0.001, ns: no significance, compared with sham group

Table S7. Statistical analysis of the primary results in LTP recording

fEPSP slope (% of baseline)

	n1	n2	n3	n4	n5
Sham	213.89±78.92	218.68±72.16	218.72±71.94	238.56±78.21	217.34±77.48
down-M	220.18±79.49	229.93±84.92	217.67±76.39	220.42±77.99	220.41±79.43
up-M	135.01±32.2	167.49±40.33	185.36±52.16	171.7±42.25	164.47±38.34

Tukey's multiple comparisons test	Mean Diff.	95.00% CI of diff.	Summary	Adjusted P Value
Sham vs. down-M	-0.2829	-3.435 to 2.869	ns	0.98
Sham vs. up-M	56.63	53.48 to 59.78	***	<0.001

Changes of fEPSP slope (mV/ms) under different stimulation intensity (microA)

	n1	n2	n3	n4	n5
Sham	4.47±3.14	4.83±3.4	5.25±3.85	4.04±2.8	3.88±2.88
down-M	4.11±2.93	4.95±3.67	4.76±3.39	3.51±2.34	3.48±2.51
up-M	1.67±1.23	2.45±2.03	2.62±2.06	1.47±1.14	1.12±0.8

Tukey's multiple comparisons test	Mean Diff.	95.00% CI of diff.	Summary	Adjusted P Value
Sham vs. down-M	0.3338	-0.02883 to 0.6965	ns	0.08
Sham vs. up-M	2.629	2.266 to 2.992	***	<0.001

Paired pulse ratio

	n1	n2	n3	n4	n5
Sham	1.42±0.15	1.2±0.13	1.35±0.12	1.32±0.12	1.36±0.14
down-M	1.28±0.13	1.26±0.11	1.48±0.15	1.24±0.1	1.28±0.12
up-M	0.94±0.04	0.92±0.07	0.92±0.06	0.92±0.06	0.94±0.05

Tukey's multiple comparisons test	Mean Diff.	95.00% CI of diff.	Summary	Adjusted P Value
Sham vs. down-M	0.02134	-0.01622 to 0.05889	ns	0.37
Sham vs. up-M	0.4022	0.3646 to 0.4397	***	<0.001
